# Cyclophilin J PPIase Inhibitors Derived from 2,3-Quinoxaline-6 Amine Exhibit Antitumor Activity

**DOI:** 10.3389/fphar.2018.00126

**Published:** 2018-02-21

**Authors:** Xuemei Zhao, Chengcai Xia, Xiaodan Wang, Hao Wang, Ming Xin, Long Yu, Yulong Liang

**Affiliations:** ^1^College of Pharmacy, Taishan Medical University, Tai’an, China; ^2^State Key Laboratory of Genetic Engineering, Institute of Genetics, School of Life Sciences, Fudan University, Shanghai, China; ^3^Michael E. DeBakey Department of Surgery, Baylor College of Medicine, Houston, TX, United States

**Keywords:** PPIL3, cyclophilin J, quinoxaline derivative, hepatocellular carcinoma, cyclophilin J inhibitor, peptidylprolyl isomerase, CyPJ, PPIase

## Abstract

Cyclophilin J (CyPJ), also called peptidylprolyl isomerase like 3, has been identified as a novel member of the cyclophilin family. Our previous research has resolved the three-dimensional structure of CyPJ and demonstrated the peptidylprolyl *cis*–*trans* isomerase (PPIase) activity of CyPJ, which can be inhibited by the common immunosuppressive drug cyclosporine A (CsA). Importantly, CyPJ is upregulated in hepatocellular carcinoma (HCC) and promotes tumor growth; CyPJ inhibition by CsA- or siRNA-based knockdown results in a remarkable suppression of HCC. These findings suggest that CyPJ may be a potential therapeutic target for HCC, and discovery of relevant inhibitors may facilitate development of a novel CyPJ-based targeting therapy. However, apart from the common inhibitor CsA, CyPJ has yet to be investigated as a target for cancer therapy. Here, we report structure-based identification of novel small molecule non-peptidic CyPJ inhibitors and their potential as antitumor lead compounds. Based on computer-aided virtual screening, *in silico*, and subsequently surface plasmon resonance analysis, 19 potential inhibitors of CyPJ were identified and selected for further evaluation of PPIase CyPJ inhibition *in vitro*. Thirteen out of 19 compounds exhibited notable inhibition against PPIase activity. Among them, the compound **ZX-J-19**, with a quinoxaline nucleus, showed potential for tumor inhibition; thus, we selected it for further structure–activity optimization. A total of 22 chemical derivatives with 2,3-substituted quinoxaline-6-amine modifications were designed and successfully synthesized. At least 2 out of the 22 derivatives, such as **ZX-J-19j** and **ZX-J-19l**, demonstrated remarkable inhibition of tumor cell growth, comparable to CsA but much stronger than 5-fluorouracil. These results indicate that these two small molecules represent novel potential lead compounds for CyPJ-based antitumor drug development.

## Introduction

Cyclophilins constitute a superfamily of peptidylprolyl isomerases (PPIase), which catalyze the *cis*–*trans* isomerization of peptide bonds on the N-terminal side of proline residue (Harikishore and Yoon, 2015; Dunyak and Gestwicki, 2016). The cyclophilin family, which is comprised of more than 15 members, was named for its ability to bind the common immunosuppressive drug cyclosporine A (CsA) (Walsh et al., 1992). Cyclophilins have been shown to act as chaperones accelerating protein folding and maturation, as well as playing a critical role in signal transduction and the immune response (Gothel and Marahiel, 1999; Wang and Heitman, 2005; Lucke and Weiwad, 2011; Harikishore and Yoon, 2015). They have also been implicated in many pathological processes, including viral infection (Towers et al., 2003; Hopkins and Gallay, 2015; Dawar et al., 2017), rheumatoid arthritis (Kim et al., 2005; Pap, 2005), cardiovascular diseases (Satoh et al., 2009; Nigro et al., 2011; Xue et al., 2017), and cancers (Lee and Kim, 2010; Nigro et al., 2013; Wang et al., 2017).

Cyclophilin J (CyPJ), also known as peptidylprolyl isomerase like 3 (PPIL3), is a new member of the cyclophilin family, with human CyPJ being first reported in 2001 (Zhou et al., 2001). CyPJ is encoded by the *CyPJ* gene initially identified in the fetal human brain. Its protein sequence exhibits 50% identity to human cyclophilin A and 72% identity to cyclophilin isoform 10 of *Caenorhabditis elegans* (Zhou et al., 2001).

Previously, we reported, for the first time, the three-dimensional structure of human CyPJ alone and in complex with CsA (Huang et al., 2005). We also identified its PPIase activity, which could be inhibited by CsA (Chen et al., 2015). The CyPJ protein contains four helices and one β-barrel composed of eight antiparallel β-strands. It also harbors a disulfide bridge and four segments with conformations strikingly different from those of CyPA (Huang et al., 2005). Specifically, His43, Arg44, and Gln52 are conserved active site residues located in the shallow pocket of CyPJ (Huang et al., 2005). Furthermore, a conserved water molecule is observed close to His43 and Gln52, while Arg44 is involved in hydrogen bonding interactions with CsA, which accounts for the observed inhibitory qualities of CsA on the PPIase activity within CyPJ (Chen et al., 2015). Importantly, we demonstrated that CyPJ is frequently upregulated in a small cohort of hepatocellular carcinoma (HCC), and CyPJ-based targeting leads to tumor suppression of HCC in a xenograft mouse tumor model (Chen et al., 2015). Several studies also report that CyPJ is upregulated in human glioma (Qi et al., 2005), estrogen receptor-negative breast cancer (Couch et al., 2016), and primary gastric adenocarcinoma (Gong et al., 2017), suggesting that the CyPJ enzyme may be an important and potential therapeutic target. However, apart from the common inhibitor of cyclophilins, CsA, CyPJ is yet to be investigated as a molecular target for cancer therapy.

A quinoxaline is an important class of nitrogen-containing heterocycle and this class of compounds plays an important role in drug development due to their potent pharmacological activity (Supplementary Table S1). For instance, marketed drugs, such as Echinomycin, Levomycin, Actinoleutin, Varenicline, Brimonidine, and Quinacillin, all contain a quinoxaline ring. In addition, quinoxaline derivatives have been reported as core motifs in medicinally active ingredients, acting as anti-protozoal, anti-proliferative, anti-inflammatory, anti-bacterial, anti-viral agents, and inhibitors of Jnk stimulatory phosphatase-1 (JSP-1), a special member of the dual-specificity protein phosphatase family 1 (Supplementary Table S1) (Hui et al., 2006; Zhang et al., 2007; Chen et al., 2011). More recently, evidence has emerged that quinoxaline-derived compounds might be capable of inhibiting tumor growth (Supplementary Table S1) (El Aissi et al., 2014; Gu et al., 2017; Miller et al., 2017). However, quinoxaline-based lead compounds have not been evaluated as inhibitors specific for target protein CyPJ to be applied in cancer therapy.

In this study, we aimed to identify novel potential inhibitors of CyPJ and characterize their possible antitumor activity, especially for HCC. To this end, we used structure-based *in silico* virtual screening (VS) followed by surface plasmon resonance and a PPIase inhibition assay to identify CyPJ inhibitors. Based on those results, we optimized antitumor activity of CyPJ inhibitors using quinoxaline nucleus-associated rational design. The evidence derived from the current study provides novel potential CyPJ inhibitors and highlights the potent inhibitory effects of these compounds on tumor cell growth.

## Materials and Methods

### General Information

Expression and purification of recombinant human CyPJ protein was carried out as described previously (Chen et al., 2015). CyPJ protein concentration was determined using the Bradford method. The substrates *N*-succinyl-Ala-Ala-Pro-Phe-*p*-nitroanilide (Suc-AAPF-pNA), CsA, 2,2,2-trifluoroethanol, and α-chymotrypsin were purchased from Sigma (St. Louis, MO, United States). The libraries of small molecular compounds used for VS were obtained from SPECS (200,000 compounds)^1^ and China Natural Products Database (CNPD, 50,000 compounds)^2^. The small organic compounds selected after VS were purchased from SPECS (Zoetermeer, Netherlands). Chemicals and solvents were either purchased from Sigma or purified by standard techniques. Analytical thin-layer chromatography (TLC) was performed on a Merck pre-coated TLC plate (silica gel 60 F_254_). Melting points were recorded on an X_4_-Data microscopic melting point apparatus and were uncorrected. Electrospray ionization-mass spectrometry (ESI-MS) was performed on a Bruker Esquire 3000 plus spectrometer. ^1^H NMR spectra were recorded on a Bruker Avance 400 spectrometer in CDCl_3_ using tetramethylsilane (TMS) as the internal standard. Elemental analysis was performed on Carlo-Erba 1106 (Carlo Erba, Italy).

### Molecular Docking and Active Site Analysis

Previously, we obtained two crystal structures of CyPJ: the CyPJ/CsA complex at 2.4 Å (PDB: 2OJU) and the CyPJ crystal alone at 2.0 Å (PDB: 2OK3) (Chen et al., 2015). In order to better mimic the affinity between CyPJ and the relevant compounds, and accurately calculate their docking scores, we selected the crystal structure of the CyPJ/CsA complex (2OJU) for molecular docking analysis. Arg44, Gln52, Asn92, His110, and Tyr115 were conserved active site residues located in the shallow pocket of the protein structure, where Arg44 displayed a flexible conformation. Our compounds of interest were then each flexibly docked on the protein structure. In our docking calculation, potential energy maps of the receptor were calculated using default parameters.

### *In Silico* Virtual Screening

Virtual screening *in silico* was carried out as described elsewhere with minor modifications (Li et al., 2006b). The structure of CyPJ/CsA complex (PDB: 2OJU) was used as a target. The DOCK 4.0 program suite was employed for primary screening of the small molecule databases SPECS and CNPD via 64CPU-SGI ORIGIN3800 (State Key Laboratory of computer-aided drug design, Shanghai Institute of Materia Medica, Chinese Academy of Sciences, Shanghai, China) and 392CPU-Shenwei I supercomputer (Shanghai Supercomputing Center) (Kuntz, 1992; Butler et al., 2016; Choi et al., 2017). Based on the X-ray structure of the CyPJ/CsA complex (Chen et al., 2015), Arg44, Gln52, Asn92, His110, and Tyr115 residues surrounding CsA were selected to generate a cavity. Our small molecules were then docked into this cavity, and ligand-binding quality was evaluated using a force-field scoring function. During the docking calculation, Kollman united-atom charges were assigned to the CyPJ protein, and Gasteiger–Marsili partial charges were assigned to the small molecules in the databases. Conformational flexibility of the compounds from the databases was considered during the docking search. Three thousand top-scoring compounds obtained by DOCK search were rescored by using the Consensus Score (CScore) method encoded in Sybyl6.8 (Sybyl molecular modeling package, version 6.8) (Houston and Walkinshaw, 2013; Campagna-Slater et al., 2014; Weill et al., 2014). Molecules with a CScore of ≥4 were visually analyzed. Finally, 74 compounds with the lowest energy and most favorable ligand orientation were selected.

### Surface Plasmon Resonance (SPR) Assay

The binding affinity of the selected compounds to CyPJ was measured by SPR with a Biacore 3000 instrument (Biacore AB Corporation, Uppsala, Sweden) as previously described (Guo et al., 2005; Chen et al., 2007). Briefly, recombinant human CyPJ protein was immobilized on an activated sensor chip via amine coupling. CyPJ protein (10 μM) was coupled to a CM5 sensor chip surface (a carboxymethylated dextran surface; CM5 chip from Biacore, Inc., Piscataway, NJ, United States) in a buffer containing 10 mM sodium acetate (pH 4.0) using standard amine coupling chemistry according to the manufacturer’s instructions. Flow cell 1 (FC-1) was used as the control surface and flow cell 2 (FC-2) contained 9000 resonance units (RU) of CyPJ (1 RU corresponds to 1 pg of protein per mm^2^). The buffer stream was passed through FC-1 and FC-2 of the bi-channel at a flow rate of 40 μl/min. Compounds to be tested were purchased from SPECS (Netherlands). All chemicals were of the highest purity available, as certified by the vendor. All compounds were stocked in 100% dimethylsulfoxide at 10 mM and diluted at graded concentrations (0.3–10 μM) with HBS-EP buffer (0.01 M HEPES buffer, pH 7.4, containing 0.15 M NaCl, 3.4 mM EDTA, and 0.005% surfactant P20) containing 1% dimethylsulfoxide. The temperature of the instrument was set to 20°C, and the flow rate was set to 40 μl/min. Each compound (50 μl) was injected sequentially. NaOH (50 mM) was used to regenerate the surface of the CM5 chip. The 1:1 Langmuir binding model was used to determine the equilibrium dissociation constant (*K*_D_). For fast interactions, the steady-state model was used to determine *K*_D_ values. All experiments were carried out in triplicate.

### PPIase Assay

The PPIase assay was carried out with minor modifications as described previously (Kofron et al., 1991). We used an α-chymotrypsin-coupled PPIase assay, where the substrate succinyl-Ala-Ala-Pro-Phe-*p*-nitroanilide (Suc-AAPF-pNA) is first converted to the *trans* conformation by PPIase, and can then be digested by α-chymotrypsin to release chromogenic *p*-nitroanilide; the latter compound is then monitored with a spectrometer. Briefly, the assay was performed under 8°C in a 100 μl system. The Suc-AAPF-pNA was dissolved in the tetrafluoroethylene containing 480 mM of LiCl (working concentration: 3.0 mM). The α-chymotrypsin was dissolved in 1 mM HCl (working concentration: 1.7 mM). When assayed, each test compound was diluted in 94 μl of assay buffer (50 mM HEPES, 100 mM NaCl; pH 8.0 at 0°C), and then mixed with 2 μl of CyPJ solution (5 μM). After equilibrating on ice for 3 h, 2 μl of α-chymotrypsin solution and 2 μl of Suc-AAPF-pNA were added to the assay mixture, and the absorbance at the wavelength 390 nm was recorded for 20 s on a Jasco V-550 Spectrophotometer (Jasco, Inc., Easton, MD, United States). Three independent experiments were performed for each test compound and the respective half maximal inhibitory concentration (IC_50_) was calculated with OriginPro 7.5 software (OriginLab, Northampton, MA, United States).

### Synthesis of 2,3-Substituted Quinoxaline-6-Amine (Compound **3**)

Briefly, benzaldehyde or furaldehyde (0.1 mol) was catalyzed by thiamine (vitamin B1, 0.02 mol) to generate 2-hydroxy-1,2-diphenylethanone or 1,2-di(furan-2-yl)-2-hydroxyethanone, respectively. The resulting alcohol hydroxyls were oxidized by Cu(NO_3_)_2_ to generate diones **1a** and **1b** in HOAc/H_2_O (1:1, V/V) at 70°C in 6 h. The selective acylation of pyrrole was catalyzed with oxalyl chloride in carbon disulfide to generate the dione **1c** at -70°C. These 1,2-ethanedione derivatives (**1a**, **1b**, and **1c**, 0.3 mol each) were mixed with 4-nitro-*o*-phenylenediamine (0.5 mol) in AcOH (100 ml), and stirred at 110°C under normal atmosphere for 8 h. The mixture was then cooled to room temperature and poured into water (300 ml) to obtain 6-nitro-2, 3-disubstituted quinoxaline (compound **2**). A reaction bottle was charged with compound **2** (0.1 mol) and Na2S*x* (0.15 equiv) in ethanol (30 ml) under normal atmosphere. The mixture was vigorously stirred by refluxing for 12 h, and cooled to room temperature and poured into water (100 ml) after competition. The mixture was next extracted with 20 ml of EtOAc three times. The combined organic layer was washed with brine (20 ml), dried with Na_2_SO_4_, and the solvent was removed under reduced pressure. Finally, compound **3** was purified as an eluent by flash column chromatography using PE/EtOAc.

### Synthesis of 2,3-Substituted Quinoxaline-6-Amine Derivatives (**ZX-J-19** Derivatives)

The synthesis of 2,3-substituted quinoxaline-6-amine derivatives (i.e., **ZX-J-19** derivatives) was performed as described elsewhere (Chen et al., 2011; Sridevi et al., 2011). Briefly, compound **3** (20 mmol) was treated with acyl chloride (30 mmol) in the presence of pyridine at room temperature in acetone. After compound **3** disappeared on TLC, the reaction mixture was poured into the water (50 ml), immediately cooled to room temperature, filtered, and purified to collect **ZX-J-19** derivatives by flash column chromatography.

### Cell Lines and MTT-Based Cell Proliferation Assay

Human HCC cells SK-HEP1 and QGY; breast cancer cells HCC1954, BT474, and MDA-MB468; ovarian cancer cells SKOV3; and prostate cancer cells PC3 and LNCaP were purchased from the Cell Bank of Chinese Academy of Sciences (Shanghai, China), and cultured in T75 flasks with complete DMEM or RPMI 1640 medium (GIBCO, Invitrogen, Gaithersburg, CA, United States) supplemented with 10% FBS (Hyclone, New Zealand), and 1% penicillin/streptomycin (Solarbio, Beijing, China) at 37°C in a humidified atmosphere containing 5% CO_2_.

Cell proliferation was determined using methylthiazolyl diphenyl-tetrazolium bromide assay (MTT; Sigma–Aldrich). The cells were seeded onto 96-well plates in a total volume of 150 μl at a density of 3.5 × 10^4^ cells/well. After incubation for 24 h, cells were treated with the selected compounds or positive control drugs 5-fluorouracil (5-FU) and CsA at the indicated dosages (0, 1.0, 5.0, 10, 50, and 250 μM). To avoid the effect of the solvent, the concentration of DMSO was less than 0.1% (v/v) in all experiments. After incubation at 37°C for 48 h, 10 μl of MTT solution (5 mg/ml) was added and continued to incubate for 4 h. Following removal of medium containing MTT, 200 μl of DMSO was added to dissolve the formazan crystals formed by live cells. Solution absorbance was measured at 490 nm with an absorbance microplate reader (BioTek, Winooski, VT, United States). The assay was repeated six times. The IC_50_ value was determined from plot of % viability against the dose of compounds added.

### Statistical Analysis

The compound activities and MTT-based cell assays were repeated at least three times and the significance was assessed with unpaired Student’s *t*-test. The survival curve was created by the Kaplan–Meier method and analyzed by the log-rank test. A *P* < 0.05 was considered statistically significant.

## Results

### Identification of Small Molecule Inhibitors of CyPJ by Virtual Screening and SPR Analysis

**FIGURE 1:**
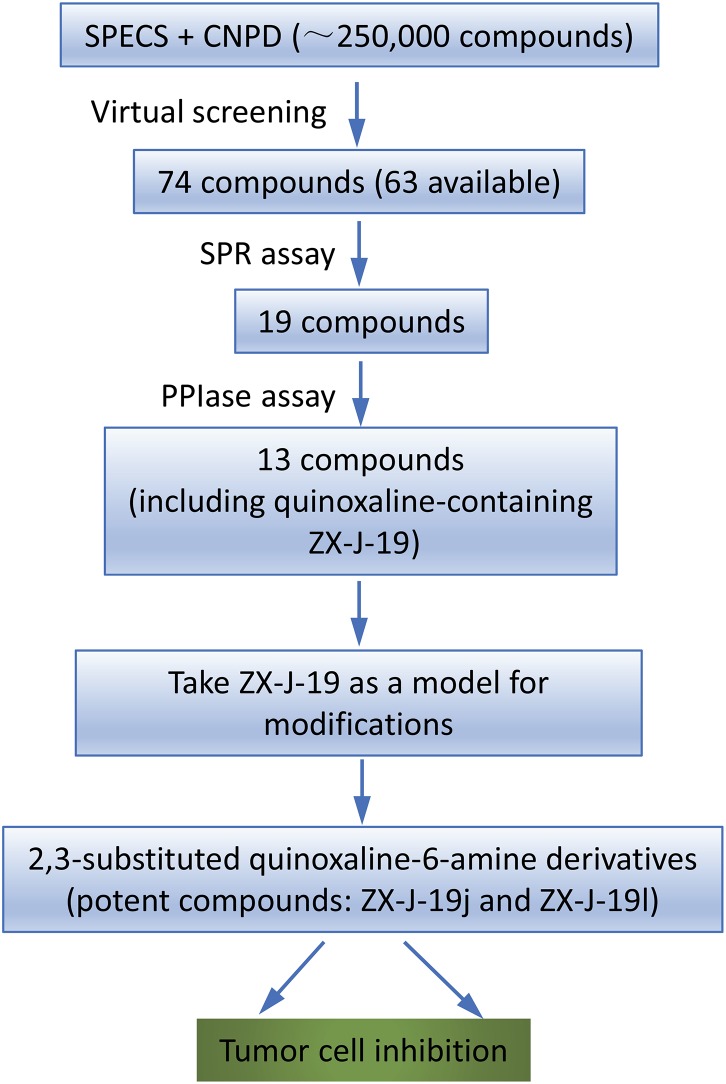
Flow chart of virtual screening and identification of CyPJ inhibitors. SPECS and CNPD are chemical libraries containing 200,000 and 50,000 compounds, respectively. SPR, surface plasmon resonance. PPIase, peptidylprolyl *cis*–*trans* isomerase.

To identify the inhibitory small molecules of CyPJ, first VS was conducted, *in silico*, using commercially available compound libraries. Crystal structures of CyPJ complexed with CsA were analyzed for potential inhibitory ligands (Huang et al., 2005; Chen et al., 2015). As illustrated in **Figure 1**, we screened SPECS and CNPD databases with a total of 250,000 small molecule compounds to determine their ability to dock at the catalytic site of CyPJ. We selected 74 compounds with the best docking scores. Among them, 63 compounds were commercially available and purchased from SPECS and their CyPJ binding was subsequently analyzed by SPR.

**FIGURE 2:**
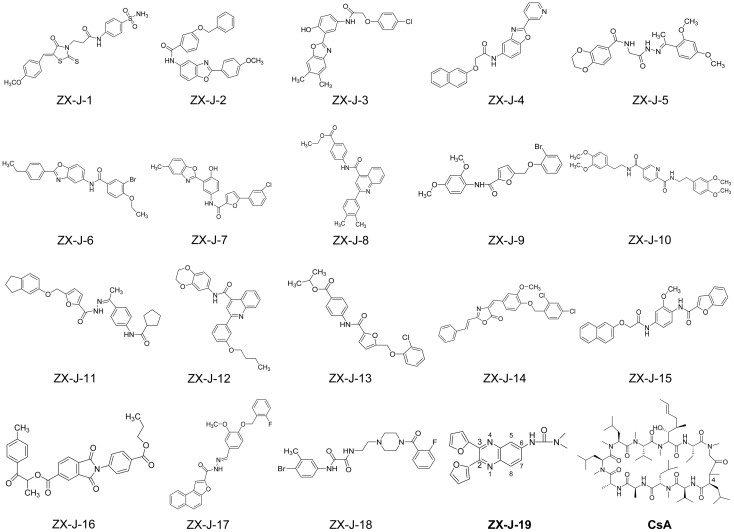
Chemical structures of 19 potential CyPJ inhibitors identified with *in silico* screening and SPR analysis. Following *in silico* screening and SPR analysis, 19 **ZX-J** compounds numbered **1–19** were identified and selected for further investigation. The numbers in the compound **ZX-J-19** represent the positions of the carbons in the quinoxaline ring. CsA, cyclosporine A, is a well-established cyclophilin protein inhibitor.

Based on SPR analysis, 19 out of 63 compounds, i.e., **ZX-J-1** to **ZX-J-19** (**Figure 2**), were found to be capable of binding with CyPJ (**Table 1**). The binding of the above 19 compounds behaved in a concentration-dependent manner with *K*_i_ values ranging from 1 × 10^-4^ M to 9 × 10^-9^ M by VS, and with *K*_D_ values ranging from 1 × 10^-4^ M to 9 × 10^-8^ M by SPR analysis (**Table 1**). These results suggested that these 19 compounds strongly interact with CyPJ.

**Table 1:** Affinity of inhibitors to CyPJ, and their respective IC_50_ for CyPJ PPIase inhibition.

Compound	Molecular weight (Da)	CyPJ binding by virtual screening *K*_i_ (M)^a^	CyPJ binding by SPR *K*_D_ (M)^b^	Compound IC_50_ for PPIase (μM)^c^
ZX-J-1	477.584	1.30 × 10^-6^	6.50 × 10^-6^	15.949 ± 0.062^∗∗^
ZX-J-2	450.492	8.38 × 10^-7^	1.35 × 10^-6^	8.944 ± 0.027^∗∗^
ZX-J-3	422.866	8.03 × 10^-8^	1.06 × 10^-6^	–^d^
ZX-J-4	395.416	3.54 × 10^-9^	1.05 × 10^-7^	11.376 ± 0.768^∗∗^
ZX-J-5	413.428	7.45 × 10^-7^	1.37 × 10^-6^	21.324 ± 0.911^∗∗∗^
ZX-J-6	465.345	1.26 × 10^-9^	2.85 × 10^-5^	17.883 ± 0.585^∗∗^
ZX-J-7	444.872	3.28 × 10^-8^	1.26 × 10^-7^	5.815 ± 0.613^∗∗^
ZX-J-8	424.498	9.41 × 10^-9^	1.45 × 10^-5^	10.346 ± 0.442^∗^
ZX-J-9	432.268	4.76 × 10^-8^	1.29 × 10^-6^	8.542 ± 0.965^∗^
ZX-J-10	493.557	2.19 × 10^-6^	8.00 × 10^-7^	–^d^
ZX-J-11	485.581	3.79 × 10^-7^	9.88 × 10^-8^	19.949 ± 0.025^∗∗∗^
ZX-J-12	454.523	1.62 × 10^-8^	1.18 × 10^-4^	12.952 ± 0.054^∗∗∗^
ZX-J-13	413.855	8.88 × 10^-8^	5.16 × 10^-7^	12.634 ± 0.068^∗∗^
ZX-J-14	480.345	1.80 × 10^-7^	1.12 × 10^-6^	–^d^
ZX-J-15	466.491	9.66 × 10^-8^	6.61 × 10^-7^	–^d^
ZX-J-16	499.517	1.01 × 10^-4^	3.41 × 10^-6^	18.456 ± 0.727^∗∗^
ZX-J-17	468.482	2.29 × 10^-7^	4.35 × 10^-8^	–^d^
ZX-J-18	491.359	5.48 × 10^-9^	2.42 × 10^-7^	–^d^
ZX-J-19	348.000	2.01 × 10^-6^	1.67 × 10^-5^	15.949 ± 0.012^∗∗^
CsA	1202.600	n.d.	n.d.	4.929 ± 0.254

*^a^K_i_, inhibitor constant. ^b^K_D_, dissociation constant. ^c∗^P < 0.05, ^∗∗^P < 0.01, ^∗∗∗^P < 0.001 for IC_50_ (μM) compared to CsA. ^d^No inhibitory effects. CsA, cyclosporine A; n.d., not determined.*

### Inhibition of the PPIase Activity by 13 Identified Compounds

To investigate whether the selected compounds inhibit the PPIase activity of CyPJ, we performed a PPIase assay as described previously (Chen et al., 2015). The inhibitory effect of each compounds was determined with a standard spectrophotometric method in chymotrypsin-coupled assays with different concentrations of peptide substrate. The rate constants for the *cis*–*trans* conversion were evaluated by fitting the data to the integrated first-order rate equation through nonlinear least-square analysis. Since CyPJ belongs to the cyclophilin protein family, the inhibitory effect of these compounds appears to follow Michaelis–Menten kinetics (Chen et al., 2015). There were 13 compounds including **ZX-J-1**, **ZX-J-2**, **ZX-J-4**, **ZX-J-5**, **ZX-J-6**, **ZX-J-7**, **ZX-J-8**, **ZX-J-9**, **ZX-J-11**, **ZX-J-12**, **ZX-J-13**, **ZX-J-16**, and **ZX-J-19** that had IC_50_ values ranging from 5 to ∼20 μM, slightly higher than that of CsA (**Table 1**). Meanwhile, no inhibition of PPIase activity of CyPJ was detectable in the remaining six compounds, **ZX-J-3**, **ZX-J-10**, **ZX-J-14**, **ZX-J-15**, **ZX-J-17**, and **ZX-J-18** (**Table 1**). We concluded that 13 out of 19 compounds may be regarded as the inhibitors of CyPJ.

### HCC Tumor Cell Growth Inhibition by Quinoxaline-Containing Compound **ZX-J-19**

Among the 13 inhibitory compounds identified previously, **ZX-J-19** is a quinoxaline derivative composed of a quinoxaline nucleus with a furanyl group at positions 2 and 3 and a urea group at position 6 (**Figure 2**). Since the quinoxaline nucleus may potentially have antitumor activity (Supplementary Table S1), together with our previous observation that siRNA-based CyPJ targeting inhibited tumor growth of HCC (Chen et al., 2015), we wanted to know whether the quinoxaline-containing **ZX-J-19** can inhibit HCC tumor cell growth. First of all, we verified the alterations of *CyPJ* in HCC in a large cohort from The Cancer Genome Atlas (TCGA). We found a higher *CyPJ* gene copy number in about 15% of HCC samples (*n* = 370) (Supplementary Figure S1A). Also, the *CyPJ* alterations were positively correlated with poor disease-free survival in HCC patients (*P* = 0.0287) (Supplementary Figure S1B), suggesting that *CyPJ* is of importance in HCC.

Next, the inhibitory effects of **ZX-J-19** were determined using MTT-based *in vitro* cell proliferation assay along with CsA (positive control for inhibiting CyPJ) and 5-FU (positive control for antitumor activity) on HCC cells SK-HEP1 and QGY. As shown in **Table 2**, the IC_50_ of **ZX-J-19** on SK-HEP1 and QGY HCC cells was 40.440 and 52.438 μM, respectively. Although these IC_50_ values were slightly higher than those of CsA (10.243 and 7.902 μM, respectively), as the positive control inhibitor of CyPJ, they were strikingly lower than those of 5-FU (177.238 and 238.528 μM, respectively), a conventional clinical drug (**Table 2**), suggesting that **ZX-J-19** potently inhibits HCC tumor cell growth *in vitro*.

**Table 2:** IC_50_ of selected compounds on HCC cells.

Entry	Compounds	IC_50_ (μM)	
		SK-HEP1	QGY
1	ZX-J-19	40.440 ± 0.033	52.438 ± 0.019^a,b^
2	5-FU	177.238 ± 0.065	238.528 ± 0.035
3	CsA	10.243 ± 0.082	7.902 ± 0.022

*^a^P < 0.05 compared to CsA. ^b^P < 0.05 compared to 5-FU.*

### Modifications of 2,3-Substituted-Quinoxaline-6-Amine Derivatives with Synthetic Chemistry

**FIGURE 3:**
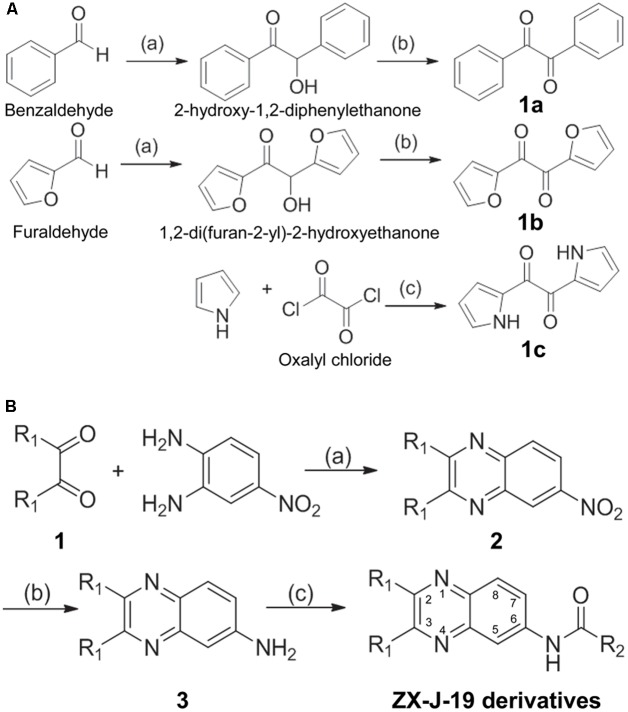
The synthesis route of 2,3-substituted-quinoxaline-6-amine derivatives. **(A)** The synthetic production of ethanediones (including intermediate chemicals **1a**, **1b**, **1c**). Reagents and conditions: (a) Vitamin B1 (VB1), EtOH/H_2_O 2:1, NaOH, room temperature, 12 h; (b) Cu(NO_3_)_2_, HOAc/H_2_O 1:1, 70°C, 6 h; (c) carbon disulfide, –70°C, pyridine, 4 h. **(B)** The synthetic process of 2,3-substituted-quinoxaline-6-amine derivatives (i.e., **ZX-J-19** derivatives). Compound **1** was referred to as **1a**, **1b**, or **1c** obtained from **(A)**. Reagents and conditions: (a) HOAc, reflux, 8 h; (b) Na_2_S*x*, *x* = 2–6, EtOH, reflux, 12 h; (c) RCOCl, pyridine, acetone, reflux, 4 h. The numbers in the **ZX-J-19** derivatives represent the position of the carbons in the quinoxaline ring. R_1_ refers to the side residuals at the positions 2 and 3 of the quinoxalines. R_2_ refers to the side residual linked to the amide group at the position 6 of the quinoxalines.

To identify more potent quinoxaline-containing compounds and further optimize them for tumor cell inhibition, we modified the quinoxaline nucleus at positions 2, 3, and 6 based on the structure of **ZX-J-19** using the rational-design strategy. First, we synthesized the ethanediones (compound **1**, e.g., **1a**, **1b**, and **1c**) as presented in **Figure 3A**, which provided the necessary diversity of the R_1_ residues at positions 2 and 3. Benzaldehyde and furaldehyde reactions were catalyzed by thiamine to generate 2-hydroxy-1,2-diphenylethanone and 1,2-di (furan-2-yl)-2-hydroxyethanone, respectively (yields: 88 and 84%, respectively). The resulting hydroxyethanone derivatives were then oxidized by Cu(NO_3_)_2_ to generate the diones **1a** and **1b** in HOAc/H_2_O (1:1, V/V) at 70°C in 6 h (yields: 91 and 87%, respectively). For the synthesis of **1c**, selective acylation of pyrrole was catalyzed with oxalyl chloride in carbon disulfide at -70°C (yield: 81%).

Next, 2,3-substituted-6-amine derivatives of quinoxaline were synthesized, and the synthesis route is illustrated in **Figure 3B**. The compounds **1a**, **1b**, or **1c** were condensed with 4-nitro-*o*-phenylene diamine in HOAc under reflux for about 8 h to generate 6-nitro-2,3-diphenyl quinoxaline, 2,3-di(furan-2-yl)-6-nitroquinoxaline, or 6-nitro-2,3-di(1H-pyrrol-2-yl) quinoxaline (compound **2**), respectively. The resulting nitro-quinoxalines (**2**) (e.g., 6-nitro-2,3-diphenyl quinoxaline) was reduced by Na_2_S*x* (1.5 equiv) under reflux in ethanol to furnish the 2,3-diphenylquinoxaline-6-amine (compound **3**). Compound **3** (1 equiv) was then treated with acyl chloride (1.3 equiv) in acetone at room temperature to produce the 22 2,3-quinoxaline-6-amine (**ZX-J-19**) derivatives, as shown in **Table 3** (**ZX-J-19a–ZX-J-19v**; yield: 63–81%). All synthesized compounds were structurally characterized by ^1^H NMR, elemental analysis, and mass spectral analysis (see the Supplementary Structure Data listed in the Supplementary Materials). These compounds were categorized into three groups according to the property of the residues at the R_1_ position: the phenyl type (**ZX-J-19a**–**ZX-J-19l**), the furanyl type (**ZX-J-19m**–**ZX-J-19s**), and the pyrrole type (**ZX-J-19t–ZX-J-19v**), and the compounds with the furanyl type were more closely related to compound **ZX-J-19**.

**Table 3:** Isolated yields of 2,3-substituted quinoxaline-6-amine derivatives.

ZX-J-19 derivatives^a^	R_1_^b^	R_2_^c^	Isolated yield (%)
a	Phenyl	-CH_2_-Ph	63
b	Phenyl	-C(Me)_3_	71
c	Phenyl	-(*p*-nitrophenyl)	65
d	Phenyl	-CH_2_Cl	79
e	Phenyl	-(*m*-chlorophenyl)	64
f	Phenyl	-(*o*-chlorophenyl)	67
g	Phenyl	-(*p*-chlorophenyl)	73
h	Phenyl	-Ph	81
i	Phenyl	-C_2_H_5_	78
j	Phenyl	-C_7_H_15_	63
k	Phenyl	-CH_3_	74
l	Phenyl	-C_3_H_7_	76
m	2-Furanyl	-C(Me)_3_	73
n	2-Furanyl	-C_3_H_7_	70
o	2-Furanyl	-C_7_H_15_	67
p	2-Furanyl	-(*o*-chlorophenyl)	65
q	2-Furanyl	-(*p*-nitrophenyl)	63
r	2-Furanyl	-Ph	80
s	2-Furanyl	-CH_2_-Ph	77
t	2-Pyrrole	-(*p*-chlorophenyl)	65
u	2-Pyrrole	-CH_3_	69
v	2-Pyrrole	-CH_2_Cl	71

*^a^A total of 22 compounds (**ZX-J-19a-ZX-J-19v**) isolated. ^b^Two R_1_ groups residing at positions 2 and 3 of the quinoxaline ring, respectively. ^c^R_2_ group was linked to the amide at position 6 of the quinoxaline ring. Ph, phenyl; Me, methyl.*

### Remarkable Inhibition of Tumor Cell Growth by **ZX-J-19** Derivatives

To determine the effects of the 22 **ZX-J-19** derivatives (**a–v**) on tumor cell growth, we first examined their activity on HCC cells as detected by the MTT-based *in vitro* cell proliferation assay. As shown in **Table 4**, four compounds **ZX-J-19e**, **ZX-J-19g**, **ZX-J-19j**, and **ZX-J-19l**, all belonging to the phenyl type of 2,3-substituted quinoxaline-6-amine derivatives, exhibited remarkable inhibitory effects on HCC cell growth. Their potency was comparable to the potency of the positive CyPJ inhibitor control, CsA, shown in **Table 2**. Among these four compounds, **ZX-J-19j** and **ZX-J-19l** exhibited the most potency. This result is evident by their lower IC_50_ on SK-HEP1 cells (6.725 and 3.512 μM, respectively) (**Table 4**), suggesting that **ZX-J-19j** and **ZX-J-19l** may be better than **ZX-J-19** itself and other **ZX-J-19** derivatives in HCC cell growth inhibition.

**Table 4:** IC_50_ of ZX-J-19 derivatives on HCC cells.

Entry	ZX-J-19 derivatives	IC_50_ (μM)^a^	
		SK-HEP1	QGY
1	a	387.602 0.044	208.271 0.021
2	b	21.595 0.031	25.976 0.009
3	c	719.724 0.036	37.644 0.079
4	d	11.350 0.013	17.943 0.033
5	e	7.495 0.057	12.314 0.0146
6	f	321.356 0.087	541.697 0.0297
7	g	9.890 0.047	11.263 0.088
8	h	40.332 0.024	27.505 0.035
9	i	40.767 0.068	588.584 0.087
10	j	6.725 0.023	12.524 0.097
11	k	7.534 0.021	198.752 0.005
12	l	3.512 0.022	21.353 0.037
13	m	76.744 0.017	82.343 0.007
14	n	17.650 0.082	18.022 0.015
15	o	17.334 0.041	98.437 0.017
16	p	45.724 0.052	548.538 0.042
17	q	43.648 0.021	44.258 0.023
18	r	18.830 0.044	17.315 0.015
19	s	332.354 0.064	416.804 0.027
20	t	46.348 0.033	42.458 0.008
21	u	14.248 0.039	32.669 0.019
22	v	140.343 0.028	127.490 0.045

*^a^IC_50_ values were determined using a MTT-based cell proliferation assay.*

Besides HCC, the *CyPJ* gene is also aberrantly dysregulated in several other malignancies such as breast, prostate, and ovarian cancers (Supplementary Figure S2) (Qi et al., 2005; Couch et al., 2016; Gong et al., 2017). With this fact in mind, we further determined if **ZX-J-19j** and **ZX-J-19l** could inhibit growth of tumor cells originating from other cancers, including breast cancer cells HCC1954, BT474, and MDA-MB468; ovarian cancer cells SKOV3; and prostate cancer cells PC3 and LNCaP. As shown in **Table 5**, both **ZX-J-19j** and **ZX-J-19l** showed significant growth inhibition in these tumor cells, suggesting **ZX-J-19j** and **ZX-J-19l** might inhibit tumor cell growth in a wide spectrum of malignancies.

**Table 5:** Growth inhibition of multiple cancer cell lines with compounds **ZX-J-19j** and **ZX-J-19l**.

Cell lines		% Growth inhibition at 20 μM	
		ZX-J-19j	ZX-J-19l
HCC1954	Breast cancer	64.177	91.258
BT474		84.007	90.963
MDA-MB-468		84.653	90.849
SKOV3	Ovarian cancer	48.067	90.919
PC3	Prostate cancer	84.653	91.039
LNCaP		48.067	91.038

It should be pointed out that although **ZX-J-19j** and **ZX-J-19l** demonstrated convincing inhibitory effects on tumor cell growth by using *in vitro* cell models, these two compounds still retain similar lipophilicity to **ZX-J-19**. This characteristic may contribute to poor antitumor activity of **ZX-J-19** in an *in vivo* xenograft mouse tumor model due to its poor pharmacokinetics (unpublished data). For example, this compound was absorbed and accumulated in blood serum within 10 min after intraperitoneal administration, peaked within 30–60 min, and then declined and diminished to control levels after 8 h. These results suggest that these 2,3-substituted quinoxaline-6 amine compounds need to be further optimized for *in vivo* application, a finding which is under investigation.

Taken together, our data suggest that these quinoxaline-containing derivatives (e.g., **ZX-J-19j** and **ZX-J-19l**) are lead compounds for novel CyPJ-targeting antitumor drugs.

## Discussion

Based on the three-dimensional structure of CyPJ reported in our previous research (Hu et al., 2005; Huang et al., 2005; Chen et al., 2015), we virtually screened the chemical libraries of SPECS and CNPD *in silico*, and were able to identify 13 inhibitors of CyPJ (**Table 1**). Because of the well-characterized potential antitumor activity of quinoxaline derivatives and their relative ease of synthesis, we focused on the quinoxaline-containing compound **ZX-J-19** and quinoxaline-based modifications of that template. A total of 22 2,3-substituted-quinoxaline-6-amine derivatives were successfully synthesized using a structure–activity relationship (SAR) strategy (**Table 3**). Moreover, we were able to provide convincing evidence that the growth in several tumor cell lines was inhibited by **ZX-J-19j** and **ZX-J-19l** (**Tables 4**, **5**). These representatives of strong antitumor activity may act as lead compounds to facilitate the development of promising drugs targeting CyPJ-associated cancers.

In this study, we confirmed aberrations of the *CyPJ* gene previously reported in HCC (Chen et al., 2015) and demonstrated that its expression was associated with HCC progression (Supplementary Figure S1). Although *CyPJ* was relatively uninvestigated previously, a few reports suggested that it might play an important role in tumorigenesis and/or progression in HCC (Chen et al., 2015) and other cancers (Qi et al., 2005; Couch et al., 2016; Gong et al., 2017). For example, we have previously shown the importance of *CyPJ* expression in HCC; CyPJ inactivation by CsA- or siRNA-based *CyPJ* knockdown diminished HCC tumor cell growth *in vitro* and *in vivo*, demonstrating CyPJ was capable of initiating tumorigenesis of HCC (Chen et al., 2015). Our observations in this study are consistent with these previous findings (Supplementary Figure S1). Previous research provided support for the further investigation of CpPJ inhibitors **ZX-J-19j** and **ZX-J-19l** as potent inhibitors of tumor cell growth.

Cyclosporine A is a well-established potent inhibitor of cyclophilins that can also block the PPIase catalytic sites of CyPJ (Chen et al., 2015). CsA has profound implications in immunosuppression for organ transplantation (Colombo and Ammirati, 2011; Ziaei et al., 2016), however, as an antitumor agent, CsA may not be suitable due to potent side effects related to secondary skin cancers and other malignancies (Behnam et al., 2005; Gallagher et al., 2010; Norman et al., 2010; Han et al., 2012; Muellenhoff and Koo, 2012). It is therefore necessary to identify novel small molecule antitumor compounds that target cyclophilins including CyPJ. In this report, we identified at least 13 compounds, including the quinoxaline-containing compound **ZX-J-19**, that exhibited notable inhibitory activities of PPIase in CyPJ (**Table 1**). Intriguingly, based on the quinoxaline nucleus scaffold of **ZX-J-19**, we rationally designed and ultimately synthesized 22 **ZX-J-19**-like quinoxaline derivatives with 2,3-substituted-6-amine modifications. Two of the resulting compounds **ZX-J-19j** and **ZX-J-19l** had notably enhanced antitumor activities (**Tables 4**, **5**).

Consistent with these results, the quinoxaline nucleus is known to exhibit antitumor activity, and quinoxaline-derived compounds have been reported to have potential in cancer treatment (Zarranz et al., 2004; Amin et al., 2006; Weng et al., 2008; Pereira et al., 2015). For example, several quinoxaline-1,4-di-*N*-oxide derivatives exhibited considerable antitumor activity in MCF-7 (breast), NCI-H460 (lung), and SF-268 (CNS) cells; such antitumor activity was dependent on the substituents at the carbonyl group (Zarranz et al., 2004). With regard to **ZX-J-19j** and **ZX-J-19l**, the phenyl at position R_1_ and the heptyl (**ZX-J-19j**) or propyl (**ZX-J-19l**) at position R_2_ were considered as the residues that contribute to the amelioration of antitumor capability. Similarly, our data indicated that at least **ZX-J-19j** and **ZX-J-19l** are optimal lead candidate compounds for further development of novel CyPJ inhibitors and the potential antitumor drugs for CyPJ-based targeting.

As discussed previously, the quinoxaline derivative structures were determined based on CyPJ protein structure. However, we cannot rule out the possibility that these derivatives may impair the PPIase activity of other human cyclophilins. There are at least 15 members of the cyclophilin protein family (Fruman et al., 1994; Galat and Metcalfe, 1995). Due to high degree of similarity between members, cyclophilins inhibitors may have overlapping inhibitory action against many cyclophilins. For instance, a potent, well-characterized cyclophilin inhibitor, CsA, can inhibit the PPIase activities of almost all cyclophilins including CyPJ (Handschumacher et al., 1984; Chen et al., 2015). Cyclophilin CyPA has similarly been reported to be involved in many diseases; CyPA PPIase-based inhibitor Debio-025 (Alisporivr) has been shown to inhibit several other cyclophilins including CyPB and CyPD (Flisiak et al., 2008; Quarato et al., 2012; Lee, 2013). Specifically for quinoxaline-derived compounds, it has been reported that they may inhibit at least CyPA and CyPD (Guo et al., 2005; Li et al., 2006a). In this respect, it may be not unexpected that our CyPJ inhibitors may also inhibit other members of cyclophilins at some extent, and this issue will be the focus of future work.

In summary, we carried out a computer-aided virtual inhibitor screening of chemical databases based on the crystal structure of CyPJ/CsA complex, and identified 13 potential inhibitors of CyPJ. The quinoxaline-containing **ZX-J-19**, and its derivatives **ZX-J-19j** and **ZX-J-19l** showed potent antitumor activity. To the best of our knowledge, this study is the first to identify the inhibitors of CyPJ; these compounds are potential leads for rational design of novel CyPJ-based antitumor drugs.

## Author Contributions

XZ and LY designed the project. XZ, CX, XW, HW, MX, and YL performed the experiments and data analysis. XZ, CX, and YL wrote the manuscript. All authors discussed the results and revised the manuscript.

## Conflict of Interest Statement

Two patents related to this work have been granted in China: one for the compound 1,2-di(1*H*-pyrrol-2-yl)ethane-1,2-dione in 2011 (Patent No. ZL200910019140.2, inventors: XZ and CX) and the other for **ZX-J-19j** [*N*-(2,3-diphenylquinoxalin-6-yl)octanamide] in 2013 (Patent No. ZL201110178176.2, inventors: XZ and CX). The other authors declare that the research was conducted in the absence of any commercial or financial relationships that could be construed as a potential conflict of interest.

## Footnotes

1 http://www.specs.net">http://www.specs.net

2 http://www.neotrident.com
