# Supplementary material for: Cyclophilin J PPIase Inhibitors Derived from 2,3-Quinoxaline-6 Amine Exhibit Antitumor Activity
Source: Front Pharmacol. 2018 Feb 21;9:126. doi: 10.3389/fphar.2018.00126 (PMC5826973; doi:10.3389/fphar.2018.00126)
Supplement: Supplementary file 1 [file Presentation_1.PDF]

Supplementary Materials

PPIase protein cyclophilin J inhibitors derived from 2,3-quinoxaline-6 amine exhibit antitumor activity

Xuemei Zhao[1#], Chengcai Xia[1#], Xiaodan Wang[1], Hao Wang[1], Ming Xin[1], Long Yu[2*], Yulong Liang[1,3*]

***Corresponding authors:**
Yulong Liang: yulong@bcm.edu
Long Yu: longyu@fudan.edu.cn

1. SUPPLEMENTARY TABLE

Supplementary Table S1. List of biological active quinoxaline-containing compounds identified in the last decade

Compounds	Activities	References
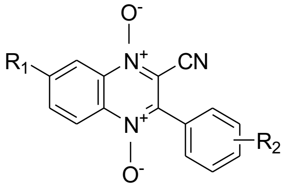	Antitumor	1
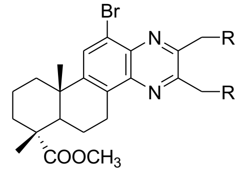	Antitumor	2
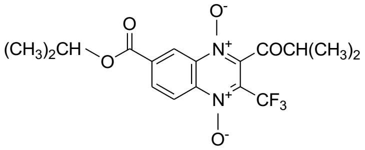	Trypanothione reductase inhibitors (IC_{50}=2.42 ± 0.5)	3
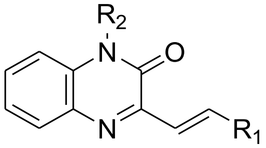	Antitumor	4
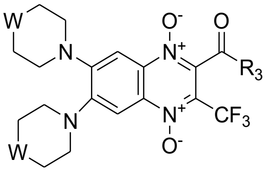	Treatment of multidrug-resistant and latent tuberculosis	5
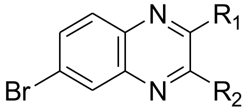	Antitumor and antimicrobial	6

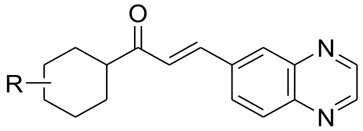	Inhibitors of breast cancer	7
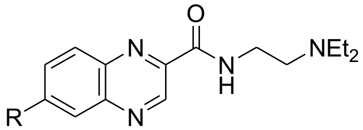	Melanoma-targeting probes	8
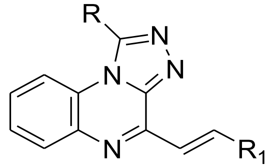	Anticonvulsant	9
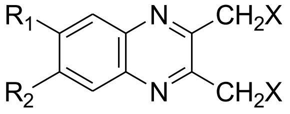	Antibacterial and antifungal	10
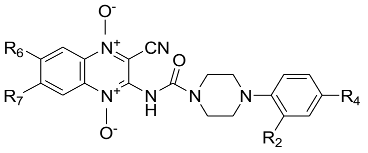	Antiplasmodial and leishmanicidal	11
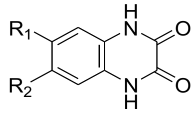	Neuropharmacological activities	12
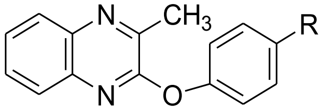	Antimicrobial	13
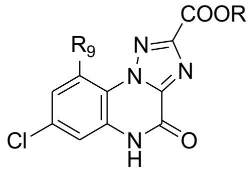	(*R,S*)-2-amino-3-(3-hydroxy-5-methylisoxazol-4-yl)propionic acid (AMPA) receptor antagonists	14
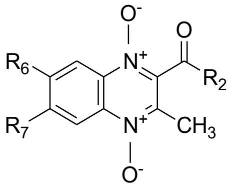	Antimycobacterial	15
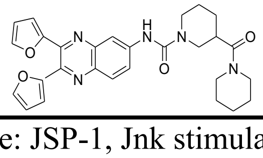	JSP-1 inhibitor (2.25 μM)	16

Note: JSP-1, Jnk stimulatory phosphatase-1.

2. SUPPLEMENTARY FIGURES

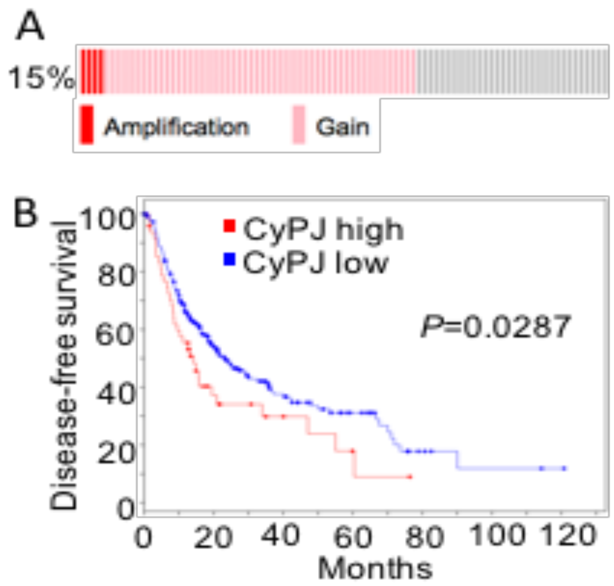

Supplementary Figure S1. The *CyPJ* gene is frequently altered in HCC samples. (A) The *CyPJ* gene was gained or amplified in 15% of TCGA HCC samples (n=370). The data were retrieved from the cBioPortal for Cancer Genomics (17, 18) as of September 21, 2017. (B) High copy number of the *CyPJ* gene was positively correlated with poor disease-free survival in the above TCGA cohort (n=370).

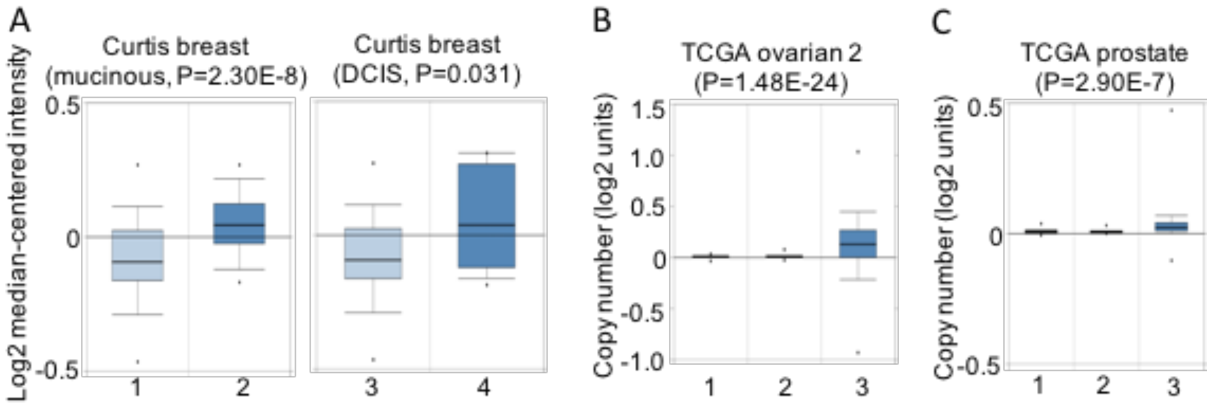

Supplementary Figure S2. *CyPJ* is altered in breast, ovarian, and prostate cancers. (A) The *CyPJ* gene expression was upregulated in breast ductal carcinoma *in situ* (DCIS) and breast mucinous carcinoma from the Curtis breast cohort (19). For the Curtis breast (mucinous), 1, normal breast (n=144); 2, mucinous breast carcinoma (n=46). For the Curtis breast (DCIS), 3, normal breast (n=144); 4, DCIS (n=10). (B and C) The copy number of the *CyPJ* gene was remarkably higher in TCGA ovarian cancer (B) and TCGA prostate cancer (C). For TCGA ovarian cancer, 1, blood (n=431); 2, ovary (n=130); 3, ovarian cystadenocarcinoma (n=607). For TCGA prostate, 1, blood (n=148); 2, prostate gland (n=61); 3, acinar prostate adenocarcinoma

(n=126). These data were retrieved from the Oncomine (www.oncomine.com) with default parameters (20).

3. SUPPLEMENTARY STRUCTURE DATA OF IDENTIFIED COMPOUNDS

_N_-(2,3-diphenylquinoxalin-6-yl)-2-phenylacetamide (ZX-J-19a)
^{1}H NMR (300 MHz, $CDCl_3$) δ: 8.54 (br.s, 1H, CONH), 8.24 (d, *J*=2.1 Hz, 1H, H-5), 8.07 (d, *J*=9.0 Hz, 1H, H-8), 7.85 (dd, *J*=9.0, 2.1 Hz, 1H, H-7), 7.28～7.50 (m, 15H, H-$PhCH_2$×5, H-diphenyl×10), 3.84 (s, 2H, H-$PhCH_2$). HRMS(ESI+): Calculated for $C_{28}H_{21}N_3O$, $[M+H]^+$416.1685. Found 416.1663.

_N_-(2,3-diphenylquinoxalin-6-yl)pivalamide (ZX-J-19b)
^{1}H NMR (300 MHz, $CDCl_3$) δ: 8.31 (d, *J*=1.5 Hz, 1H, H-5), 8.11 (d, *J*=9.1 Hz, 1H, H-8), 7.99 (dd, *J*=9.1,1.5 Hz, 1H, H-7), 7.65 (br.s, 1H, CONH), 7.50 (m, 4H, H- diphenyl-3`, 5`, 3``, 5``), 7.30 (m, 6H, H-diphenyl-2`, 4`, 6`, 2``, 4``, 6``), 1.38 (s, 9H, $(CH_3)_3CCO$). HRMS(ESI+): Calculated for $C_{25}H_{23}N_3O$, $[M+H]^+$382.1841. Found 382.1875.

_N_-(2,3-diphenylquinoxalin-6-yl)-4-nitrobenzamide (ZX-J-19c)
^{1}H NMR (300 MHz, $CDCl_3$) δ: 8.52 (br.s, 1H, CONH), 8.35 (m, 3H, H-5, H-NO_2PhCO×2), 8.12-8.21 (m, 4H, H-8, H-7, H-NO_2PhCO×2), 7.53 (m, 4H, H-diphenyl-3`, 5`, 3``, 5``), 7.35 (m, 6H, H-diphenyl-2`, 4`, 6`, 2``, 4``, 6``). HRMS(ESI+): Calculated for $C_{27}H_{18}N_4O_3$, $[M+H]^+$447.1379. Found 447.1391.

2-chloro-N-(2,3-diphenylquinoxalin-6-yl)acetamide (ZX-J-19d)
^{1}H NMR (300 MHz, $CDCl_3$) δ: 8.56 (br.s, 1H, CONH), 8.48 (br.s, 1H, H-5), 8.15 (d, *J*=9.0 Hz, 1H, H-8), 7.99 (br.d, *J*=9.0 Hz, 1H, H-7), 7.52 (m, 4H, H-diphenyl-3`, 5`, 3``, 5``), 7.34 (m, 6H, H-diphenyl-2`, 4`, 6`, 2``, 4``, 6``), 4.36 (s, $ClCH_2CO$). HRMS(ESI+): Calculated for $C_{22}H_{16}ClN_3O$, $[M+H]^+$374.0982. Found 374.0971.

3-chloro-_N_-(2,3-diphenylquinoxalin-6-yl)benzamide (ZX-J-19e)
^{1}H NMR (300 MHz, $CDCl_3$) δ: 8.77 (br.s, 1H, CONH), 8.49 (br.s, 1H, H-5), 8.8 (m, 2H, H-8, H-7), 7.82 (br.s, 1H, H-ClPhCO), 7.72 (d, *J*=7.6 Hz, 1H, H-ClPhCO), 7.26-7.47 (m, 12H, H-PhCO×2, H-diphenyl×10), 7.35 (m, 6H, H-diphenyl-2`, 4`, 6`, 2``, 4``, 6``). HRMS(ESI+): Calculated for $C_{27}H_{18}ClN_3O$, $[M+H]^+$436.1138. Found 436.1155.

2-chloro-_N_-(2,3-diphenylquinoxalin-6-yl)benzamide (ZX-J-19f)
^{1}H NMR (300 MHz, $CDCl_3$) δ: 8.48 (br.s, 1H, H-5), 8.39 (br.s, 1H, CONH), 8.16 (d, *J*=9.0 Hz, 1H, H-8), 8.07 (br.d, *J*=9.0 Hz, 1H, H-7), 7.80 (d, *J*=7.4 Hz, 1H, H-ClPhCO), 7.18-7.53 (m, 13H, H-PhCO×3, H-diphenyl×10), 7.35 (m, 6H, H-diphenyl-2`, 4`, 6`, 2``, 4``, 6``). HRMS(ESI+): Calculated for $C_{27}H_{18}ClN_3O$, $[M+H]^+$436.1138. Found 436.1161.

4-chloro-_N_-(2,3-diphenylquinoxalin-6-yl)benzamide (ZX-J-19g)
^{1}H NMR (300 MHz, $CDCl_3$) δ: 8.46 (d, *J*=2.2 Hz, 1H, H-5), 8.29 (br.s, 1H, CONH), 8.16 (d, *J*=9.0 Hz, 1H, H-8), 8.10 (dd, *J*=9.0, 2.2 Hz, 1H, H-7), 7.87 (d, 2H, H-ClPhCO×2), 7.49 (m, 6H, H-ClPhCO×2, H-diphenyl-3`, 5`, 3``, 5``), 7.35 (m, 6H, H-diphenyl-2`, 4`, 6`, 2``, 4``, 6``).HRMS(ESI+): Calculated for $C_{27}H_{18}ClN_3O$, $[M+H]^+$436.1138. Found 436.1127.

***N*-(2,3-diphenylquinoxalin-6-yl)benzamide (ZX-J-19h)**

^{1}H NMR (300 MHz, $CDCl_3$) δ: 8.47 (br.s, 1H, H-5), 8.38 (br.s, 1H, CONH), 8.10 (m, 2H, H-8, H-7), 7.91 (m, 2H, H-PhCO×2), 7.26-7.56 (m, 13H, H-PhCO×3, H-diphenyl×10), 7.35 (m, 6H, H-diphenyl-2`, 4`, 6`, 2``, 4``, 6``). HRMS(ESI+): Calculated for $C_{27}H_{19}N_3O$, $[M+H]^+$402.1528. Found 402.1547.

***N*-(2,3-diphenylquinoxalin-6-yl)propionamide (ZX-J-19i)**

^{1}H NMR (300 MHz, $CDCl_3$) δ: 8.31 (d, *J*=1.8 Hz, 1H, H-5), 8.10 (d, *J*=9.1 Hz, 1H, H-8), 7.99 (dd, *J*=9.1, 1.8 Hz, 1H, H-7), 7.68 (br.s, 1H, CONH), 7.50 (m, 4H, H- diphenyl-3`, 5`, 3``, 5``), 7.34 (m, 6H, H-diphenyl-2`, 4`, 6`, 2``, 4``, 6``), 2.48 (q, *J*=7.7 Hz, 2H, CH_3CH_2CO), 1.29 (t, *J*=7.7 Hz, 3H, CH_3CH_2CO). HRMS(ESI+): Calculated for $C_{23}H_{19}N_3O$, $[M+H]^+$354.1528. Found 354.1557.

***N*-(2,3-diphenylquinoxalin-6-yl)octanamide (ZX-J-19j)**

^{1}H NMR (300 MHz, $CDCl_3$) δ: 8.30 (d, *J*=1.8 Hz, 1H, H-5), 8.11 (d, *J*=9.0 Hz, 1H, H-8), 7.99 (br.d, *J*=9.0 Hz, 1H, H-7), 7.53 (br.s, 1H, CONH), 7.50 (m, 4H, H- diphenyl-3`, 5`, 3``, 5``), 7.34 (m, 6H, H-diphenyl-2`, 4`, 6`, 2``, 4``, 6``), 2.45 (t, *J*=7.4 Hz, 2H, $CH_3CH_2CH_2CH_2CH_2CH_2CH_2CO$), 1.61 (br.s, 4H, $CH_3CH_2CH_2CH_2CH_2CH_2CH_2CO$), 1.25 (br.s, 6H, $CH_3CH_2CH_2CH_2CH_2CH_2CH_2CO$), 0.87 (t, *J*=7.4 Hz, 3H, $CH_3CH_2CH_2CH_2CH_2CH_2CH_2CO$). HRMS(ESI+): Calculated for $C_{28}H_{29}N_3O$, $[M+H]^+$424.2311. Found 424.2337.

***N*-(2,3-diphenylquinoxalin-6-yl)acetamide (ZX-J-19k)**

^{1}H NMR (300 MHz, $CDCl_3$) δ: 8.33 (br.s, 1H, H-5), 8.11 (d, *J*=9.1 Hz, 1H, H-8), 8.00 (dd, *J*=9.1, 1.8 Hz, 1H, H-7), 7.61 (br.s, 1H, CONH), 7.47(m, 4H, H- diphenyl-3`, 5`, 3``, 5``), 7.31 (m, 6H, H-diphenyl-2`, 4`, 6`, 2``, 4``, 6``), 2.28 (s, 3H, CH_3CO). HRMS(ESI+): Calculated for $C_{22}H_{17}N_3O$, $[M+H]^+$340.1372. Found 340.1394.

***N*-(2,3-diphenylquinoxalin-6-yl)butyramide (ZX-J-19l)**

^{1}H NMR (300 MHz, $CDCl_3$) δ: 8.31 (br.s, 1H, H-5), 8.10 (d, *J*=9.0 Hz, 1H, H-8), 7.99 (br.d, *J*=9.0 Hz, 1H, H-7), 7.64 (br.s, 1H, CONH), 7.50 (m, 4H, H- diphenyl-3`, 5`, 3``, 5``), 7.30 (m, 6H, H-diphenyl-2`, 4`, 6`, 2``, 4``, 6``), 2.43 (t, *J*=7.4 Hz, 2H, $CH_3CH_2CH_2CO$), 1.81 (sext, *J*=7.4 Hz, 2H, $CH_3CH_2CH_2CO$), 1.04 (t, *J*=7.4 Hz, 3H, $CH_3CH_2CH_2CO$). HRMS(ESI+): Calculated for $C_{24}H_{21}N_3O$, $[M+H]^+$368.1785. Found 368.1766.

***N*-(2, 3-di(furan-2-yl)quinoxalin-6-yl) pivalamide (ZX-J-19m)**

^{1}H NMR (300 MHz, DMSO-d_6) δ 9.70 (br.s, NH), 8.57 (d, J = 1.7 Hz, 1H, H-5), 8.08 (dd, J = 9.3, 2.3 Hz, 1H, H-7), 8.01 (d, J = 8.9 Hz, 1H, H-8), 7.88, (m, 2H, H-furanyl-5`, 5``), 6.70 (m, 4H, H- furanyl-3`, 4`, 3``, 4``),1.30 (s, 9H, CH_3×3). HRMS(ESI+): Calculated for $C_{21}H_{19}N_3O_3$, $[M+H]^+$362.1426. Found 362.1451.

***N*-(2,3-di(furan-2-yl)quinoxalin-6-yl)butyramide (ZX-J-19n)**

^{1}H NMR (300 MHz, DMSO-d_6) δ 10.43 (br.s, NH), 8.53 (d, J = 2.1 Hz, 1H, H-5), 8.03 (d, J = 9.1 Hz, 1H, H-8), 7.90 (dd, J = 8.9, 2.3 Hz, 1H, H-7), 7.87 (m, 2H, H-furanyl-5`, 5``), 6.69 (m, 4H, H-furanyl-3`, 4`, 3``, 4``), 2.41 (t, J = 7.4 Hz, 2H, -$CH_2CH_2CH_3$), 1.67 (m, 2H, -

$CH_2CH_2CH_3$), 0.96 (t, J = 7.1 Hz, 3H, -$CH_2CH_2CH_3$). HRMS(ESI+): Calculated for $C_{20}H_{17}N_3O_3$, $[M+H]^+$348.1270. Found 348.1279.

***N*-(2,3-di(furan-2-yl)quinoxalin-6-yl)octanamide (ZX-J-19o)**
^{1}H NMR (300 MHz, DMSO-d_6) δ 10.43 (br.s, NH), 8.53 (d, J = 2.1 Hz, 1H, H-5), 8.02 (d, J = 9.1 Hz, 1H, H-8), 7.90 (dd, J = 9.1, 2.1 Hz, 1H, H-7), 7.87 (m, 2H, H-furanyl-5`, 5``), 6.69 (m, 4H, H-furanyl-3`, 4`, 3``, 4``), 2.42 (t, J = 7.4 Hz, 2H, -$CH_2CH_2(CH_2)_4CH_3$), 1.65 (m, 2H, -$CH_2CH_2(CH_2)_4CH_3$), 1.30 (m, 8H, -$CH_2CH_2(CH_2)_4CH_3$), 0.96 (t, J = 6.9, 3H, -$CH_2CH_2(CH2)_4CH_3$). HRMS(ESI+): Calculated for $C_{24}H_{25}N_3O_3$, $[M+H]^+$404.1896. Found 404.1917.

2-chloro-*N*-(2,3-di(furan-2-yl)quinoxalin-6-yl)benzamide (ZX-J-19p)
^{1}H NMR (300 MHz, DMSO-d_6) δ 11.09 (br.s, N*H*), 8.64 (d, J = 1.3 Hz, 1H, H-5), 8.10 (d, J = 9.2 Hz, 1H, H-8), 8.04 (dd, J = 9.1, 1.9 Hz, 1H, H-7), 7.89 (m, 2H, H-furanyl-5`, 5``), 7.70 (dd, J = 7.4, 1.7 Hz, 1H, H-Ph-6```), 7.61 (dt, J = 7.2, 1.7 Hz, 1H, H-Ph-4```), 7.55 (dd, J = 7.8, 1.8 Hz, 1H, H-Ph-3```), 7.51 (dt, J = 7.3, 1.6 Hz, 1H, H-Ph-5```), 6.72 (m, 4H, H-furanyl-3`, 4`, 3``, 4``). HRMS(ESI+): Calculated for $C_{23}H_{14}ClN_3O_3$, $[M+H]^+$416.0727. Found 416.0755.

***N*-(2,3-di(furan-2-yl)quinoxalin-6-yl)-4-nitrobenzamide (ZX-J-19q)**
^{1}H NMR (300 MHz, DMSO-d_6) δ 11.07 (br.s, NH), 8.70 (d, J = 1.4 Hz, 1H, H-5), 8.43 (d, J = 8.8 Hz, 2H, H-Ph-3```, 5```), 8.27 (d, J = 8.8, Hz, 1H, H-Ph-2```, 6```), 8.17 (dd, J = 9.1, 1.9 Hz, 1H, H-7), 8.11 (d, J = 8.7 Hz, 1H, H-8), 7.90 (m, 2H, H-furanyl-5`, 5``), 6.74 (m, 4H, H-furanyl-3`, 4`, 3``, 4``). HRMS(ESI+): Calculated for $C_{23}H_{14}N_4O_5$, $[M+H]^+$427.0964. Found 427.0947.

***N*-(2,3-di(furan-2-yl)quinoxalin-6-yl)benzamide (ZX-J-19r)**
^{1}H NMR (300 MHz, DMSO-d_6) δ 10.79 (br.s, NH), 8.70 (d, J = 2.3 Hz, 1H, H-5), 8.18 (dd, J = 9.2, 2.3 Hz, 1H, H-7), 8.08 (d, J = 9.0 Hz, 1H, H-8), 8.03 (d, J = 6.8, 1.6 Hz, 2H, H-Ph-2```, 6```), 7.90 (m, 2H, H-furanyl-5`, 5``), 7.61 (m, 3H, H-Ph-3```, 4```, 5```), 6.72 (m, 4H, H-furanyl-3`, 4`, 3``, 4``). HRMS(ESI+): Calculated for $C_{23}H_{15}N_3O_3$, $[M+H]^+$382.1113. Found 382.1136.

***N*-(2,3-di(furan-2-yl)quinoxalin-6-yl)-2-phenylacetamide (ZX-J-19s)**
^{1}H NMR (300 MHz, DMSO-d_6) δ 10.74 (br.s, NH), 8.51(d, J = 2.1 Hz, 1H, H-5), 8.04 (d, J = 9.0 Hz, 1H, H-8), 7.92 (dd, J = 8.9, 2.2 Hz, 1H, H-7), 7.88 (m, 2H, H-furanyl-5`, 5``), 7.33 (m, 5H, H-Ph-2```, 3```, 4```, 5```), 6.69 (m, 4H, H-furanyl-3`, 4`, 3``, 4``), 3.77 (s, 2H, -$COCH_2Ph$). HRMS(ESI+): Calculated for $C_{24}H_{17}N_3O_3$, $[M+H]^+$396.1270. Found 396.1287.

4-chloro-*N*-(2,3-di(1H-pyrrol-2-yl)quinoxalin-6-yl)benzamide(ZX-J-19t)
^{1}H NMR (300 MHz，$CDCl_3$) δ: 9.73 (br.s, 1H, -pyrrol-NH), 9.53 (br.s, 1H, -pyrrol-NH), 8.21 (br.s, 1H, H-5), 8.17(br.s, 1H, CONH), 7.82 (m, 3H, H-8, H-ClPhCO×2), 7.47 (m, 3H, H-7, H-ClPhCO×2), 6.90 (m, 4H, H-dipyrrol-2`, 5`, 2``, 5``), 6.27 (m, 2H, H-dipyrrol-4`, 4``); ESI-MS *m/z*: 414.2 ($[M+H]^+$), 849.1 ($[2M+Na]^+$), 412.3 ($[M-H]^-$). HRMS(ESI+): Calculated for $C_{23}H_{16}ClN_5O$, $[M+H]^+$414.1043. Found 414.1061.

***N*-(2,3-di(1H-pyrrol-2-yl)quinoxalin-6-yl)acetamide (ZX-J-19u)**
^{1}H NMR (300 MHz, $CDCl_3$) δ: 9.78 (br.s, 1H, -pyrrol-NH), 9.59 (br.s, 1H, -pyrrol-NH), 8.13 (d, *J*=2.5 Hz, 1H, H-5), 7.79 (d, *J*=9.2 Hz, 1H, H-8), 7.64(br.d, *J*=9.2 Hz, 1H，H-7), 7.50 (br.s, 1H,

CONH), 6.90 (m, 4H, H-dipyrrol-2`, 5`, 2``, 5``), 6.26 (m, 2H, H-dipyrrol-4`, 4``), 2.17 (s, 3H, CH_3CO); ESI-MS *m/z*: 318.2 ($[M+H]^+$), 340.1 ($[M+Na]^+$), 657.1 ($[2M+Na]^+$), 316.0 ($[M-H]^-$). HRMS(ESI+): Calculated for $C_{18}H_{15}N_5O$, $[M+H]^+$318.1277. Found 318.1289.

2-chloro-N-(2,3-di(1H-pyrrol-2-yl)quinoxalin-6-yl)acetamide (ZX-J-19v)
^1H NMR (300 MHz, $CDCl_3$) δ: 9.75 (br.s, 1H, -pyrrol-NH), 9.59 (br.s, 1H, -pyrrol-NH), 8.47 (br.s, 1H, CONH), 8.25 (d, *J*=2.5 Hz, 1H, H-5), 7.85 (d, *J*=9.3 Hz, 1H, H-8)，7.64(dd, *J*=9.3, 2.5 Hz, 1H, H-7)，6.98 (m, 4H, H-dipyrrol-2`, 5`, 2``, 5``)，6.28 (m, 2H, H-dipyrrol-4`, 4``), 4.22 (s, 2H, H-$ClCH_2CO$); HRMS(ESI+): Calculated for $C_{18}H_{14}ClN_5O$, $[M+H]^+$352.0887. Found 352.0899.

4. SUPPLEMENTARY REFERENCES

1. Miller EM, Xia Q, Cella ME, Nenninger AW, Mruzik MN, Brillos-Monia KA, Hu YZ, Sheng R, Ragain CM, Crawford PW. Voltammetric Study of Some 3-Aryl-quinoxaline-2-carbonitrile 1,4-di-N-oxide Derivatives with Anti-Tumor Activities. Molecules. 2017 Aug 31;22(9). pii: E1442. doi: 10.3390/molecules22091442.
2. Gu W, Wang S, Jin X, Zhang Y, Hua D, Miao T, Tao X, Wang S. Synthesis and Evaluation of New Quinoxaline Derivatives of Dehydroabietic Acid as Potential Antitumor Agents. Molecules. 2017 Jul 11;22(7). pii: E1154. doi: 10.3390/molecules22071154.
3. Chacón-Vargas KF, Nogueda-Torres B, Sánchez-Torres LE, Suarez-Contreras E, Villalobos-Rocha JC, Torres-Martinez Y, Lara-Ramirez EE, Fiorani G, Krauth-Siegel RL, Bolognesi ML, Monge A, Rivera G. Trypanocidal Activity of Quinoxaline 1,4 Di-N-oxide Derivatives as Trypanothione Reductase Inhibitors. Molecules. 2017 Feb 1;22(2). pii: E220. doi: 10.3390/molecules22020220.
4. Liu Z, Yu S, Chen D, Shen G, Wang Y, Hou L, Lin D, Zhang J, Ye F. Design, synthesis, and biological evaluation of 3-vinyl-quinoxalin-2(1H)-one derivatives as novel antitumor inhibitors of FGFR1. Drug Des Devel Ther. 2016 May 3;10:1489-500. doi: 10.2147/DDDT.S88587. eCollection 2016.
5. Santivañez-Veliz M, Pérez-Silanes S, Torres E, Moreno-Viguri E. Design and synthesis of novel quinoxaline derivatives as potential candidates for treatment of multidrug-resistant and latenttuberculosis. Bioorg Med Chem Lett. 2016 May 1;26(9):2188-2193. doi: 10.1016/j.bmcl.2016.03.066. Epub 2016 Mar 18.
6. Al-Marhabi AR, Abbas HA, Ammar YA. Synthesis, Characterization and Biological Evaluation of Some Quinoxaline Derivatives: A Promising and Potent New Class of Antitumor and Antimicrobial Agents. Molecules. 2015 Nov 3;20(11):19805-19822. doi: 10.3390/molecules201119655.
7. Winter E, Gozzi GJ, Chiaradia-Delatorre LD, Daflon-Yunes N, Terreux R, Gauthier C, Mascarello A, Leal PC, Cadena SM, Yunes RA, Nunes RJ, Creczynski-Pasa TB, Di Pietro A. Quinoxaline-substituted chalcones as new inhibitors of breast cancer resistance protein ABCG2: polyspecificity at B-ring position. Drug Des Devel Ther. 2014 May 27;8:609-619. doi: 10.2147/DDDT.S56625. eCollection 2014.
8. El Aissi R, Liu J, Besse S, Canitrot D, Chavignon O, Chezal JM, Miot-Noirault E, Moreau E. Synthesis and Biological Evaluation of New Quinoxaline Derivatives of ICF01012 as Melan oma-Targeting Probes. ACS Med Chem Lett. 2014 Feb 20;5(5):468-473. doi: 10.1021/ml400468x. eCollection 2014 May 8.
9. Alswah M, Ghiaty A, El-Morsy A, El-Gamal K. Synthesis and Biological Evaluation of

Some [1,2,4]Triazolo[4,3-a]quinoxaline Derivatives as Novel Anticonvulsant Agents. ISRN Org Chem. 2013 Sep 12;2013:587054. doi: 10.1155/2013/587054. eCollection 2013.

10. Ishikawa H, Sugiyama T, Yokoyama A. Synthesis of 2,3-bis(halomethyl)quinoxaline derivatives and evaluation of their antibacterial and antifungal activities. Chem Pharm Bull (Tokyo). 2013;61(4):438-444.
11. Barea C, Pabón A, Galiano S, Pérez-Silanes S, Gonzalez G, Deyssard C, Monge A, Deharo E, Aldana I. Antiplasmodial and leishmanicidal activities of 2-cyano-3-(4-phenylpiperazine-1-carboxamido) quinoxaline 1,4-dioxide derivatives. Molecules. 2012 Aug 7;17(8):9451-961. doi: 10.3390/molecules17089451.
12. Jubie S, Gayathri R, Kalirajan R. Synthesis and neuropharmacological evaluation of some novel quinoxaline 2, 3-dione derivatives. Scientific World Journal. 2012;2012:718023. doi: 10.1100/2012/718023. Epub 2012 May 2.
13. Singh DP, Deivedi SK, Hashim SR, Singhal RG. Synthesis and Antimicrobial Activity of Some New Quinoxaline Derivatives. Pharmaceuticals (Basel). 2010 Jul 30;3(8):2416-2425.
14. Catarzi D, Colotta V, Varano F, Filacchioni G, Gratteri P, Sgrignani J, Galli A, Costagli C. Synthesis and biological evaluation of novel 9-heteroaryl substituted 7-chloro-4,5-dihydro-4-oxo-1,2,4-triazolo[1,5-a]quinoxaline-2-carboxylates (TQX) as (R,S)-2-amino-3-(3-hydroxy-5-methylisoxazol-4-yl)propionic acid (AMPA) receptor antagonists. Chem Pharm Bull (Tokyo). 2008 Aug;56(8):1085-1091.
15. Villar R, Vicente E, Solano B, Pérez-Silanes S, Aldana I, Maddry JA, Lenaerts AJ, Franzblau SG, Cho SH, Monge A, Goldman RC. In vitro and in vivo antimycobacterial activities of ketone and amide derivatives of quinoxaline 1,4-di-N-oxide. J Antimicrob Chemother. 2008 Sep;62(3):547-54. doi: 10.1093/jac/dkn214. Epub 2008 May 23.
16. Zhang L, Qiu B, Xiong B, Li X, Li J, Wang X, Li J, Shen J. Quinoxalinylurea derivatives as a novel class of JSP-1 inhibitors. Bioorg Med Chem Lett. 2007 Apr 15;17(8):2118-2122. Epub 2007 Feb 2.
17. Gao J, Aksoy BA, Dogrusoz U, Dresdner G, Gross B, Sumer SO, Sun Y, Jacobsen A, Sinha R, Larsson E, Cerami E, Sander C, Schultz N. Integrative analysis of complex cancer genomics and clinical profiles using the cBioPortal. Sci Signal. 2013 Apr 2;6(269):pl1. doi: 10.1126/scisignal.2004088.
18. Cerami E, Gao J, Dogrusoz U, Gross BE, Sumer SO, Aksoy BA, Jacobsen A, Byrne CJ, Heuer ML, Larsson E, Antipin Y, Reva B, Goldberg AP, Sander C, Schultz N. The cBio cancer genomics portal: an open platform for exploring multidimensional cancer genomics data. Cancer Discov. 2012 May;2(5):401-404. doi: 10.1158/2159-8290.CD-12-0095.
19. Curtis C, Shah SP, Chin SF, Turashvili G, Rueda OM, Dunning MJ, Speed D, Lynch AG, Samarajiwa S, Yuan Y, Gräf S, Ha G, Haffari G, Bashashati A, Russell R, McKinney S; METABRIC Group, Langerød A, Green A, Provenzano E, Wishart G, Pinder S, Watson P, Markowetz F, Murphy L, Ellis I, Purushotham A, Børresen-Dale AL, Brenton JD, Tavaré S, Caldas C, Aparicio S. The genomic and transcriptomic architecture of 2,000 breast tumours reveals novel subgroups. Nature. 2012 Apr 18;486(7403):346-352. doi: 10.1038/nature10983.
20. Rhodes DR, Kalyana-Sundaram S, Mahavisno V, Varambally R, Yu J, Briggs BB, Barrette TR, Anstet MJ, Kincead-Beal C, Kulkarni P, Varambally S, Ghosh D, Chinnaiyan AM. Oncomine 3.0: genes, pathways, and networks in a collection of 18,000 cancer gene expression profiles. Neoplasia. 2007 Feb;9(2):166-180.
